# Upregulation of Toll-like receptor 2 expression in colorectal cancer infected by human cytomegalovirus

**DOI:** 10.3892/ol.2014.2621

**Published:** 2014-10-15

**Authors:** XUTONG LI, DONGMENG QIAN, FANG JU, BIN WANG

**Affiliations:** 1 Department of Microbiology, Medical College, Qingdao University, Qingdao, Shandong 266071, P.R. China; 2 Department of Oncology, Second Affiliated Hospital, Medical College, Qingdao University, Qingdao, Shandong 266042, P.R. China

**Keywords:** colorectal cancer, human cytomegalovirus, IE1-72, Toll-like receptor 2

## Abstract

The aim of the present study was to investigate the association between Toll-like receptor 2 (TLR2) expression and human cytomegalovirus (HCMV) in colorectal carcinoma by detecting the expression of IE1–72, TLR2, TLR4 and tumor necrosis factor (TNF)-α in colorectal carcinoma and colon adenoma samples, as well as by analyzing the mRNA levels of the proteins in colon cancer cell lines, following HCMV infection. For this study, 56 colorectal cancer and 36 colon adenoma samples were collected, and normal mucosal tissue adjacent to the tumor was used as the control. The expression of the IE1–72, TLR2, TLR4, nuclear factor (NF)-κB and TNF-α protein was detected by immunohistochemistry. Cells from the SW480 human colon carcinoma cell line were infected with HCMV. The expression of IE1–72, TLR2, TLR4, NF-κB and TNF-α mRNA was quantified at different time points prior to and following infection. The positive expression rate of IE1–72 was 44.6% (25/56) in colorectal cancer and 41.7% (15/36) in colon adenoma. These rates were significantly higher when compared with the 12.5% (7/56) observed in the normal tissues adjacent to the cancer tissues (P<0.05). The expression levels of TLR2, TLR4, NF-κB and TNF-α in colorectal cancer and adenoma were also higher than those in the control tissues. Furthermore, the expression of IE1–72 in colorectal cancer tissues was found to correlate with TLR2 and TLR4, and the correlation coefficients were 0.515 and 0.462, respectively. Following the infection of SW480 cells, the mRNA levels of TLR2 and TNF-α increased gradually from 6 h, peaked at 48 h, and then decreased gradually. No significant differences in TLR4 and NF-κB expression were identified. The results of the present study indicated that there is a specific association between HCMV and the occurrence and development of colorectal cancer, which may be facilitated by the TLR2 signaling pathway.

## Introduction

In recent decades, cancer has become a main cause of mortality in humans. Worldwide, ~12.73 million cases of cancer occur annually, resulting in 7.6 million mortalities. Colorectal cancer is one of the most common types of gastrointestinal malignancies. Of all cancers, it has the third highest global incidence, the second highest mortality rate in developed countries (1) and the fifth highest mortality rate in China (2).

The pathogenesis of malignancy is complex and depends on the genetic, environmental, lifestyle and other factors of the host. Furthermore, infectious factors exhibit a significant influence on the development and progression of malignancy (3–6). It is clear that there is a close association between certain cancers and infection by pathogenic microorganisms, including hepatitis B, hepatitis C, *Helicobacter pylori*, *Clonorchis sinensis* and *Schistosoma mansoni*. Human cytomegalovirus (HCMV) is also considered to be closely associated with cancer (7). As the HCMV gene and protein expression can be detected in colorectal cancer tissue (8,9), it was hypothesized that HCMV infection may be associated with colorectal cancer. However, certain studies have opposed this hypothesis; the expression of HCMV in frozen tissues of colorectal carcinoma was investigated by immunohistochemistry and nested polymerase chain reaction (PCR) and negative results were demonstrated and, thus, the authors did not identify an association between HCMV and colorectal cancer (10–12). Therefore, further study is required to determine whether the occurrence and development of colorectal cancer is closely associated with HCMV and, if they are associated, the mechanism of this association also remains to be elucidated.

The present study aimed to examine the mechanism of the occurrence and development of colorectal cancer, which is associated with HCMV infection and the signal transduction pathways of TLRs. Furthermore, the expression levels of inflammatory cytokines were detected in colorectal carcinoma.

## Materials and methods

### Collection of specimens and clinical data

Samples were collected from the Department of General Surgery, the Second Affiliated Hospital of Qingdao University Medical College (Qingdao, China) between March and September 2012, which included 56 cases of colorectal cancer and 36 cases of colon adenoma. Samples of the normal mucosa adjacent to the cancerous tissue were used as controls. The cancer cases included 30 males and 26 females with a median age of 67 years (range, 46–78 years). A total of 12 cases of highly differentiated adenocarcinoma, 26 cases of moderately differentiated adenocarcinoma and 18 cases of poorly differentiated adenocarcinoma were identified. None of the patients had received chemotherapy or radiotherapy. The specimens were embedded in paraffin for immunohistochemical staining. This study was approved by the ethics committee of Qingdao University (Qingdao, China) and written informed consent was obtained from all patients.

### Immunohistochemistry

Mouse anti-human monoclonal HCMV IE1–72 antibody was purchased from Abcam (Cambridge, UK) and used at a 1:40 dilution. Rabbit anti-human polyclonal antibodies against TLR2, TLR4, NF-κB, interleukin (IL)-6, IL-8 and tumor necrosis factor (TNF)-α were purchased from Wuhan Boster Biological Engineering Co., Ltd. (Wuhan, China) and used at dilutions of 1:100. The immunohistochemistry kits were also purchased from Wuhan Boster Biological Engineering Co. Ltd.

For streptavidin-biotin complex (SABC) immunohistochemistry, the biopsy specimens were paraffin-embedded, cut into 4-μm sections, dewaxed and antigen-repaired in the microwave for 10 min. The sections were then treated with the primary antibody at 37°C for 65 min and the secondary antibody at 37°C for 20 min in a water bath. The SABC was added at 37°C for 20 min and washed with phosphate-buffered saline (PBS) four times, subsequent coloration with 3,3-diaminobenzidine for 15 min and washing with distilled water, the samples were stained with hematoxylin followed by dehydration, clearing and mounting with neutral gums and observed under a microscope (C5050, Olympus Corporation, Tokyo, Japan) PBS was used instead of primary antibody as a negative control. The tissues The positive TLR2, TLR4, NF-κB and TNF-α (Wuhan Boster Biological Engineering Co., Ltd.) were used as positive controls for TLR2, TLR4, NF-κB and TNF-α expression. IE1–72, NF-κB and TNFα localized in the cytoplasm and nucleus; TLR2 and TLR4 localized in the cytoplasm and membrane. The result was determined by the phase-weighting method based on the number of positive cells and staining intensity (14). Samples in which the number of positive cells was <5% received a score of 0, 5–24% received a score of 1, 25–49% received a score of 2, 50–74% received a score of 3, ≥75% received a score of 4. Staining intensity was scored as follows: Yellow staining, 1; brown staining, 2; and tan staining, 3. The total staining score was calculated as the sum of the percentage positivity and staining intensity scores; a total score of 1–2 was considered negative staining (−), while a total score of ≥3 was considered positive staining (+).

### Infection of SW480 cells in vitro

SW480 cells, a colon cancer cell line from the Department of Microbiology (Qingdao University), were used and the AD169 strain of HCMV was donated by the Pasteur Institute (Paris, France). Virus multiplicity of infection (MOI) was calculated as the virus plaque forming units per ml divided by the cell count. In the preliminary experiment, the majority of cells were infected when the MOI was 5. Thus, an MOI of 5 was used throughout the rest of the study.

For infection, a single cell suspension of SW40 cells was cultured in a 0.01% polylysine-coated 60-mm dish. A total of five dishes were used for the experimental group (infection group) and a control group. The infection group was treated with HCMV (MOI=5) and the control group was treated with an equivalent volume of D/F12 (200μl; 1:1) medium prior to culturing the cells for 3 days at 37°C. Morphological changes were observed with an inverted microscope (XD-101, Jiangnan Photoelectricity Co., Ltd., China) every day. Cells were then collected to extract the mRNA for reverse transcription (RT)-PCR in the pre-infection stage (0 h) and at 6, 12, 24, 48 and 72 h following infection.

### RT-PCR

Total RNA was extracted from cells using TRIzol reagent (Wuhan Boster Biological Engineering Co., Ltd.) and a UV spectrophotometer (Beijing Purkinje General Instrument Co., Ltd., Beijing, China) was used to determine RNA concentration and purity. cDNA was reverse transcribed using a reverse transcription kit [Promega (Beijing) Biotech Co., Ltd, Beijing, China] in accordance with the manufacturer’s instructions. The reaction product was preserved at −20°C. The primers were designed according to the HCMV AD l69-specific gene sequence of IE1–72 (GenBank accession no. X17403.1). The primers were synthesized by Shanghai Sangon Biological Engineering Technology & Services Co., Ltd., and the sequences are shown in Table I. RT-PCR was performed. Briefly, the target gene was amplified by PCR, using the following reaction conditions: initial denaturation at 94°C for 5 min, denaturation at 94°C for 30 sec, primer annealing at 55°C for 30 sec, elongation at 72°C for 30 sec, all for 30 cycles, final elongation at 72°C for 10 min.

The PCR products were analyzed by 1.5% agarose gel electrophoresis, and the results were observed under ultraviolet illumination. When a band clearly appeared in the appropriate size range, it was considered to be positively amplified. Images of the resulting gels were captured using the UVP gel imaging system (UVP Co. Ltd., Upland, CA, USA). Quantitative and qualitative analyses of the results were performed based on gray-scale values by the Quantity One imaging system (Bio-Rad Laboratories, Hercules, CA, USA). The gray-scale value ratio of the target gene and the internal reference, β-actin, was considered as the relative content of the mRNA of each gene. The experiment was performed in triplicate for each sample.

### Statistical analysis

Statistical analysis was performed using SPSS software, version 17.0 (SPSS, Inc., Chicago, IL, USA) to perform the χ^2^ test, Spearman’s correlation analysis. P<0.05 was considered to indicate a statistically significant difference.

## Results

### Expression of IE1–72, TLR2, TLR4, NF-κB and TNF-α in colorectal carcinoma and adenoma tissue is significantly higher than in normal mucosal tissues adjacent to cancer

**Figure 1:**
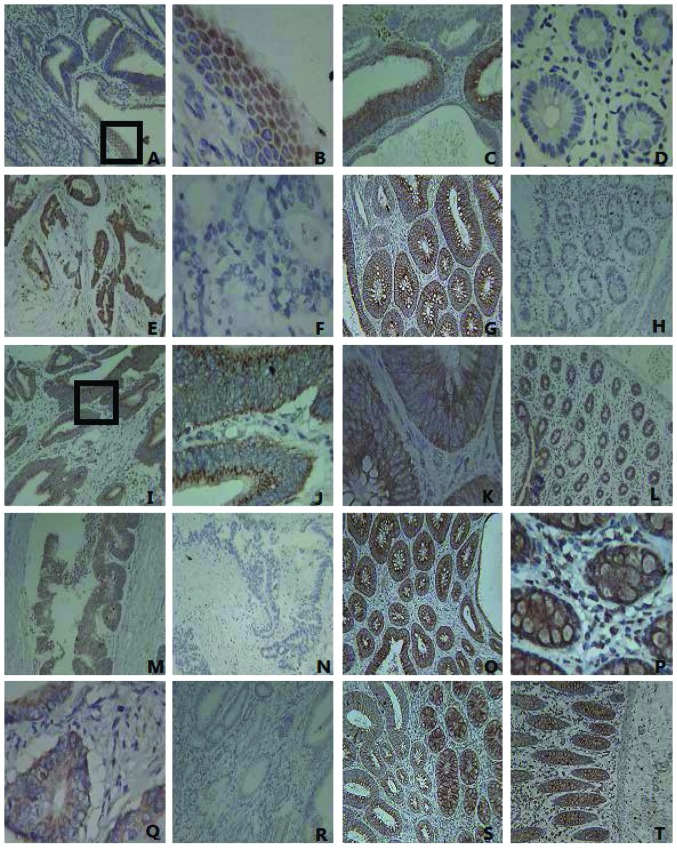
Expression of IE1–72, TLR2, TLR4, NF-κB and TNF-α in colorectal cancer tissues, adenoma and normal tissues. (A) The expression of IE1–72 in colorectal carcinomas was positive (magnification, ×100). The IE1–72 protein is visible as brown granules located in the nucleus. (B) IE1–72 expression from part A (enclosed by a black box) is shown at a higher magnification (x400). (C) The positive expression of IE1–72 was identified in adenoma, visualized as brown granules located in the cytoplasm (magnification, ×100). (D) The negative expression of IE1–72 in normal tissues (magnification, ×400). (E) The positive expression of TLR2 in colorectal carcinomas was indicated by brown particles located in the cytoplasm (magnification, ×100). (F) The negative expression of TLR2 in colorectal cancer (magnification, ×400). (G) The positive expression of TLR2 in adenoma (magnification, ×100). (H) The negative expression of TLR2 in normal tissues (magnification, ×100). (I) The positive expression of TLR4 in colorectal carcinomas (x100) was localized by brown particles in the cell membrane and cytoplasm. (J) TLR4 expression from part I (enclosed by a black box) is shown at a higher magnification (x400). (K) The positive expression of TLR4 in adenoma corresponds to the colored portions of cytoplasm and membrane (magnification, ×400). (L) The positive expression of TLR4 in normal tissues (magnification, ×100). (M) The positive expression of NF-κB in colorectal cancer tissues (magnification, ×100×). (N) The negative expression of NF-κB in colorectal cancer tissues (magnification, ×100). (O) The positive expression of NF-κB in adenoma (magnification, ×100). (P) The positive expression of NF-κB in adjacent normal tissue, where it appears as clear dark brown particles in the cytoplasm (magnification, ×400). (Q) Positive TNF-α protein expression observed mainly in the cytoplasm (brown particles) in colorectal cancer tissues (magnification ×400). (R) The negative expression of TNF-α in colorectal cancer (magnification, ×100). (S) The positive expression of TNF-α in colorectal adenoma (magnification, ×100). (T) The positive expression of TNF-α in normal tissue (magnification, ×100). TLR, Toll-like receptor; NF, nuclear factor; TNF, tumor necrosis factor.

Immunohistochemical analysis revealed that the expression of IE1–72, TLR2, TLR4, NF-κB and TNF-α protein in colorectal carcinoma was significantly higher than that in the normal tissues. The differences were statistically significant (P<0.05), and the expression levels of IE1–72, TLR4 and TNF-α in the colon adenoma were evidently higher than those in normal tissue (P<0.05) (Table II; Fig. 1). The results indicated that HCMV infection may be associated with colorectal cancer and adenoma and inflammatory responses may be mediated by the TLR2 or TLR4 signaling pathways. Therefore, further experiments at the cellular level were conducted to confirm this.

### Correlation between the protein expression of IE1–72, TLR2 and TLR4 in colorectal carcinoma

In 25 positive cases of IE1–72, there were 24 positive cases of TLR2 and 24 positive cases of TLR4 in the colorectal cancer samples. Statistical analysis of the results revealed that the correlation coefficients between IE1–72 and TLR2 and TLR4 were 0.515 and 0.462, respectively. HCMV infection was found to correlate with the expression of TLR2 and TLR4 in colorectal cancer.

### mRNA levels of TLR2 IE1–72, TLR4 and NF-κB in SW480 cells following infection

**Figure 2:**
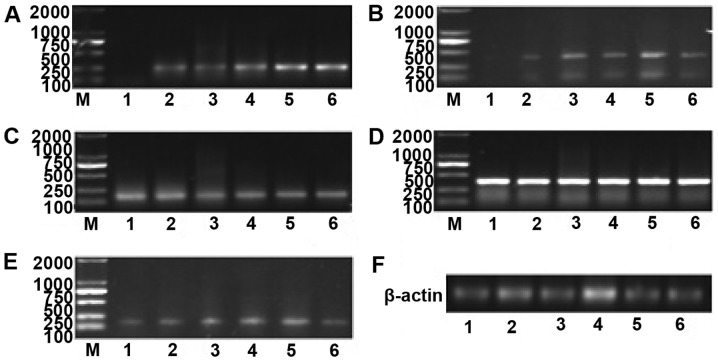
mRNA expression of IE1–72, TLR2, TLR4, NF-κB and TNF-α at various time points after SW480 cells were infected. (A) mRNA expression of IE1–72 increased in a time-dependent manner. (B) TLR2 mRNA levels were upregulated at 6 h, exhibited a prominent increase at 12 h, reached their peak at 48 h and decreased gradually after 48 h. (C)No significant differences in TLR4 mRNA were identified. (D) NF-κB mRNA was expressed at high levels, and no significant difference in expression was identified over time. (E) TNF-α mRNA began to increase at 6 h, peaked at 48 h, and decreased gradually after 48 h. (F) The internal reference gene, β-actin. 1, prior to HCMV infection (0 h); 2, 6 h after infection; 3, 12 h after infection; 4,24 h after infection; 5, 48 h after infection; and 6, 72 h after infection; M: marker. TLR, Toll-like receptor; NF, nuclear factor; TNF, tumor necrosis factor; HMCV, human cytomegalovirus.

**Figure 3:**
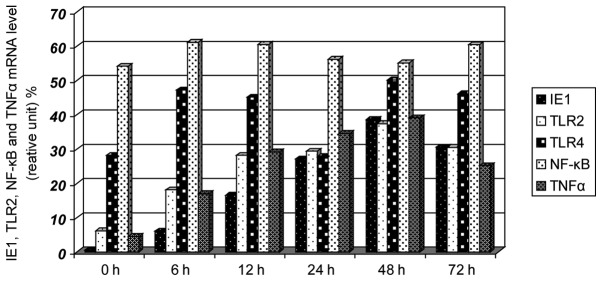
Effects on the mRNA expression levels of IE1–72, TLR2, TLR4, NF-κB and TNF-α following SW480 cell HCMV infection. After SW480 cells were infected with HCMV, the mRNA levels of TLR2 and TNF-α increased at 6 h and had increased significantly by 12 h, reaching peak levels at 48 h, the levels then decreased. TLR4 and NF-κB showed no clear changes. TLR, Toll-like receptor; NF, nuclear factor; TNF, tumor necrosis factor; HMCV, human cytomegalovirus.

Prior to infection (0 h), no expression of IE1–72 was identified, the expression levels of TLR2 and TNF-α were low, and the expression levels of TLR4 and NF-κB were high. The expression of IE1–72 increased after 6 h, reached a peak after 48 h and decreased after 72 h. The expression of TLR2 and TNF-α increased gradually with IE1–72 and reached a peak at 48 h. No significant differences in TLR4 and NF-κB expression were identified (Figs. 2 and 3). HCMV infection may upregulate the expression of TLR2 and TNF-α.

## Discussion

HCMV belongs to the β-herpes virus family and is the largest DNA virus among the human herpes viruses. The human population is generally susceptible to HCMV. In healthy individuals, HCMV exhibits the characteristics of latency-reactivation, which is an important immune escape mechanism that HCMV uses to establish a long-term coexistence process with the host. The complex interactions between HCMV and the immune system are considered to be closely associated with numerous complex diseases. Although the mechanism of occurrence and development of cancer is not clear, a number of studies have detected the expression of HCMV in different tumor tissues. The expression of the cytomegalovirus gene and protein has been detected in colorectal cancer, malignant glioma and prostate cancer and, in these cases, the adjacent normal tissues exhibited a low expression (15–17). Therefore, the authors hypothesized that HCMV may cause chronic inflammation and lead to cancer, similar to other viruses, and that HCMV is important in colon tumorigenesis development. *In vitro* studies of HCMV have also demonstrated that HCMV could induce cell malignancy and disrupt cell regulatory pathways. In addition, HCMV was found to exhibit characteristics of tumorigenicity, such as causing gene mutations and abnormal regulation of the cell cycle, inhibiting/promoting apoptosis, inducing angiogenesis, inducing cell immune escape, and regulating the synthesis and secretion of cytokines in host cells, in addition to other behaviors (18–21).

Although numerous studies have hypothesized that HCMV infection is involved in the occurrence and development of cancer, no direct evidence of the underlying mechanism has been provided. Based on the characteristics of the complex latent-reactivation of HCMV, studies have used the concepts of ‘hit and run’ and ‘tumor control’ to describe the mechanism of action of HCMV in tumorigenesis. The researchers emphasized that the genetic environment provided by tumor cells is different from that of normal cells (including transcription factors, signal transduction pathways and the dysfunction of anti-oncogenic proteins), and that this environment is advantageous for persistent infection by HCMV and exhibits a regulatory role in cancer (12,22).

The results of the present study demonstrated that the positive expression rate of IE1–72 protein in colorectal cancer and colon adenoma was 44.6 and 41.7%, respectively, which was significantly higher than the expression in normal tissues (P<0.05). These results are consistent with the results of the study by Cobbs et al (15), which demonstrated a close association between HCMV infection and the occurrence of colorectal cancer and adenomas. The results of the present study also show colon adenoma may be the early stage of precancerous lesions of the colon.

The mechanism by which HCMV enters the colonic mucosal cells remains unclear. The process by which HCMV causes chronic inflammation, leading to malignant transformation is also unclear. It has been reported that glycoproteins on cell membranes mediated and guided the process of HCMV entry into the host cell (23). Compton *et al* (13) identified a TLR, TLR2, that recognizes the capsid protein of HCMV, which further activates NF-κB and, thus, activates the innate immune response and causes the secretion of inflammatory cytokines. TLRs were identified as pathogen recognition receptors of the innate immune system; the activated signal transduction pathway of TLRs is involved in the classical MyD88 signal transduction pathway. After ligand binding, TLRs initiate activation of the NF-κB pathway and mitogen activated protein kinases pathway, resulting in the transcription and release of cytokines and inflammatory chemotactic factors. TLRs are important for the body’s resistance to the invasion of pathogenic organisms (24). It has been found that the signal transduction pathways mediated by TLRs are important in the process of tumorigenesis (25). TLR2 may rapidly recognize the envelope protein of HCMV and trigger immune responses immediately, prior to the virus entering the cells, which may provide a timing advantage in the recognition reaction resulting in cells infected by HCMV (26). TLR2 is one of the main ways for HCMV to activate innate immune responses (27). As a result of this activation, certain intracellular components of the infected cells will be altered or cell disruption occurs and cell lysates are released. Components of these lysates may be recognized by TLR2 as endogenous ligands, thus potentiating the immune response via dendritic cells and macrophages to initiate antiviral effects.

TNF-α is a critical pro-inflammatory cytokine, which is involved in cancer progression. High expression of TNF-α in the tumor microenvironment is a common characteristic of numerous malignant tumors, which are usually associated with poor prognoses. A number of studies have proposed that TNF-α is essential for carcinogenesis that is associated with inflammation (10,28,29).

In the present study, the mRNA of TLR2 and its downstream inflammatory factor, TNF-α, were upregulated with the increased expression of IE1–72 in SW480 cells following HCMV infection. This indicated that the release of chronic inflammation factors caused by infection with HCMV may be achieved via the TLR2 signaling pathway in the tumor microenvironment, thereby further inducing cell malignancy. To confirm this hypothesis, future studies are required to evaluate whether the expression of IE1–72 and TNF-α and other inflammatory factors is downregulated following the blockade of the TLR2 signaling pathway. As tumorigenesis is a long process, it is difficult to confirm a close association between viral infection and cancer. Therefore, the examination of other signaling pathways is required to investigate the effects of HCMV on malignant transformation in normal colorectal mucosa.

This study also demonstrated that the expression of TLR4 and NF-κB was increased in colorectal cancer tissue specimens; however, no significant differences in the mRNA expression of TLR4 and NF-κB were identified. NF-κB is not usually in the active state in the cytoplasm. Following activation, NF-κB translocates to the nucleus, binds its target and promotes transcription of the gene (30,31). Assessment of the activity and translocation of NF-κB is required in order to understand the underlying mechanism.

A broad and in-depth examination of the mechanism, existence and significance of HCMV in colorectal cancer may reveal novel insights with regard to the etiology of cancer. These insights may present novel strategies for the prevention of cancer and identify novel antiviral treatments for patients with HCMV, leading to the development of highly efficient vaccines and novel therapeutic approaches.

## Figures and Tables

**Table I:** Primers used in this study.

Genetic element	PCR product size	Sequence
IE1–72 (F)	251 bp	5′-GAGTCCTCTGCCAAGAGAAA-3′
IE1–72 (R)		5′-GAGTTCTGCCAGGACATCTTT-3′
TLR2 (F)	405 bp	5′-TCGGAATGTCACAGGACAGC-3′
TLR2 (R)		5′-CAGTTCATACTTGCACCACTCAC-3′
TLR4 (F)	170 bp	5′-TGAGCAGTCGTCCTGGTATC-3′
TLR4 (R)		5′-CAGGGCTTTTCTGAGTCGTC-3′
NF-κB (F)	398 bp	5′-GAAGAAGCGAGACCTGGAG-3′
NF-κB (R)		5′-TCCGGAACACAATGGCCAC-3′
TNF-α (F)	171 bp	5′-TCGGAATGCACAGGACAGC-3′
TNF-α (R)		5′-CAGTTCATACTTGCACCACTCAC-3′
β-actin (F)	154 bp	5′-TGGAACGGTGAAGGTGACAG-3′
β-actin (R)		5′-GGCTTTTAGGATGGCAAGGG-3′

TLR, Toll-like receptor; NF, nuclear factor; TNF, tumor necorsis factor; PCR, polymerase chain reaction; F, forward; R, reverse.

**Table II:** Expression of IE1–72, TLR2, TLR4, NF-κB and TNF-α in colorectal cancer, adenoma and normal tissues, which were adjacent to the cancer tissues.

	Positive expression, % (n)				
	
Tissue type	IE1–72	TLR2	TLR4	NF-κB	TNF-α
Adenocarcinoma (n=56)	44.6 (25)	69.6 (39)	73.2 (41)	80.4 (45)	80.4 (45)
Normal mucosa (n=56)	12.5 (7)	42.8 (24)	35.7 (20)	39.2 (22)	33.9 (19)
Adenoma (n=36)	41.7 (15)	55.5 (20)	83.3 (30)	83.3 (20)	66.6 (24)

TLR, Toll-like receptor; NF, nuclear factor; TNF, tumor necorsis factor.
